# Peripheral electrical stimulation to reduce pathological tremor: a review

**DOI:** 10.1186/s12984-021-00811-9

**Published:** 2021-02-15

**Authors:** Alejandro Pascual-Valdunciel, Grace W. Hoo, Simon Avrillon, Filipe Oliveira Barroso, Jennifer G. Goldman, Julio C. Hernandez-Pavon, José L. Pons

**Affiliations:** 1 Neural Rehabilitation Group, Cajal Institute, Spanish National Research Council (CSIC), Madrid, Spain; 2 E.T.S. Ingenieros de Telecomunicación, Universidad Politécnica de Madrid, Madrid, Spain; 3 Legs + Walking Lab, Shirley Ryan AbilityLab, Chicago, IL 60611 USA; 4 Department of Physical Medicine and Rehabilitation, Feinberg School of Medicine, Northwestern University, Chicago, IL USA; 5 Parkinson’s Disease and Movement Disorders, Shirley Ryan AbilityLab, Chicago, IL USA; 6 Department of Neurology, Feinberg School of Medicine, Northwestern University, Chicago, IL USA; 7 Department of Biomedical Engineering and Mechanical Engineering, McCormick School of Engineering, Northwestern University, Chicago, IL USA

**Keywords:** Tremor, Parkinson’s disease, Essential tremor, Electrical stimulation, Afferent fibers, Neural circuitry, Stimulation parameters

## Abstract

Interventions to reduce tremor in essential tremor (ET) and Parkinson’s disease (PD) clinical populations often utilize pharmacological or surgical therapies. However, there can be significant side effects, decline in effectiveness over time, or clinical contraindications for these interventions. Therefore, alternative approaches must be considered and developed. Some non-pharmacological strategies include assistive devices, orthoses and mechanical loading of the tremorgenic limb, while others propose peripheral electrical stimulation. Specifically, peripheral electrical stimulation encompasses strategies that activate motor and sensory pathways to evoke muscle contractions and impact sensorimotor function. Numerous studies report the efficacy of peripheral electrical stimulation to alter tremor generation, thereby opening new perspectives for both short- and long-term tremor reduction. Therefore, it is timely to explore this promising modality in a comprehensive review. In this review, we analyzed 27 studies that reported the use of peripheral electrical stimulation to reduce tremor and discuss various considerations regarding peripheral electrical stimulation: the stimulation strategies and parameters, electrodes, experimental designs, results, and mechanisms hypothesized to reduce tremor. From our review, we identified a high degree of disparity across studies with regard to stimulation patterns, experimental designs and methods of assessing tremor. Having standardized experimental methodology is a critical step in the field and is needed in order to accurately compare results across studies. With this review, we explore peripheral electrical stimulation as an intervention for tremor reduction, identify the limitations and benefits of the current state-of-the-art studies, and provide ideas to guide the development of novel approaches based on the neural circuitries and mechanical properties implied in tremor generation.

## Introduction

Tremor, defined as an involuntary movement of a body part or limb due to pathological neural oscillations that are projected to muscles [1], can be a hallmark of essential tremor (ET) and Parkinson’s disease (PD) and can have a debilitating effect on patients with these diagnoses. In many patients with ET and PD, tremor predominantly affects the upper extremities, typically with action or posture in ET and typically at rest in PD, and this can affect performance of daily activities [2]. Although tremors occur in both ET and PD, these disorders differ in regards to epidemiology, clinical features, and pathophysiology. ET is estimated to affect 4–5% of the population above 65 years old, while PD is estimated to affect around 1% of the population above 60 years old [1, 3]. It is thought that dysfunction of cerebello–thalamo–cortical circuitry driven by the cerebellum may underlie ET [4, 5]. ET pathophysiology further implicates the basal ganglia, cerebellum, red nucleus, and inferior olive area of the brain, i.e., the cortico–ponto–cerebello–thalamo–cortical loop and the Guillain–Mollaret triangle [6, 7]. However, the clinical diagnosis of ET is typically due to the exclusion of other pathologies causing tremor [1, 8]. PD is a progressive neurological disorder characterized pathologically by neurodegeneration including the progressive death of nigral dopaminergic cells and deposition of Lewy bodies and Lewy neurites [9]. Clinical motor symptoms of PD include tremor, predominantly at rest, along with bradykinesia/akinesia, rigidity, and gait and balance impairment. In addition, numerous non-motor features affecting mood, cognition, sleep, and autonomic systems, among others, occur in PD [2]. The classic rest tremor in PD involves the basal ganglia structures (globus pallidus interna and subthalamic nucleus) and cerebello–thalamo–cortical circuits with evidence from deep brain recordings, and neuroimaging studies [4, 5]. One hypothesis is that the basal ganglia plays a role in activating the rest tremor in PD through relationships between pallidal-cortical neurons and the cerebello–thalamo–cortical circuit functions to modulate the tremor amplitude, similar to a “dimmer-switch” [4]. Whether re-emergent tremor and postural tremor in PD result from similar pathophysiology to the characteristic classic rest tremor has not been fully elucidated. Currently there is no cure for either ET or PD. First-line or “gold standard” pharmacological treatments for tremor management for ET include propranolol and primidone [1, 10], and for PD, dopaminergic medications such as levodopa [11]. However, the use of these drug therapies may be limited by side effects, tolerance development, incomplete benefit, or for ET, the absence of efficacy as demonstrated in a large population with randomized controlled trials [1]. Alternatively, surgical interventions [12] such as deep brain stimulation (DBS) or high-intensity focused ultrasound (FUS) [13] are often considered for tremor management in ET and PD. Although DBS and FUS differ in many respects (e.g., methodology, implementation, targets, patient criteria), important drawbacks and considerations remain for these interventional therapies including possible side effects, patient selection criteria, patient interest in procedures, efficacy in treating non-tremor symptoms of PD (i.e., with DBS), reversibility (i.e., with FUS), among others [13]. Non-pharmacological strategies for tremor management have existed for years, including rehabilitation therapies, assistive devices, weighted utensils, and others, but have had limited evidence or clinical trials [14]. Interest in orthoses based on mechanical tremor reduction has grown over the years, but to date for some interventions patient perception of their use is poor and real-life applications are limited or impractical [14, 15].

Therefore, in recent years, electrical stimulation techniques at the peripheral level such as functional electrical stimulation (FES) or transcutaneous electrical nerve stimulation (TENS) have been considered as suitable interventions to reduce tremor; these techniques may bypass some of the limitations for pharmacological or surgical interventions (e.g., candidacy, side effects, among others) [16]. Given the emergence of these new therapeutic strategies, a review investigating peripheral electrical stimulation interventions for tremor reduction in ET and PD, their methodologies and their results, is timely in the field. Here, we conduct a review of the literature; discuss key elements across the studies, namely target populations, electrical stimulation approaches, stimulation parameters, experimental designs, efficiency in reducing tremor, and physiological sources of tremor reduction; and identify limitations and strengths of the studies. From the review, we then propose ways to address barriers and guide the development of new approaches based on neural circuitries and mechanical properties related to tremor generation.

## Methodology

A literature search was conducted using four databases: Scopus, Embase, PubMed, and the Institute of Electrical and Electronics Engineers (IEEE) Xplore, from date of database inception through November 9, 2020. We utilized the following search query in “title or abstract” fields to return 2396 records: "electrical stimulation" OR "electrical*" OR "nerve stimulation" OR "neuromodulation" OR "muscle stimulation" OR “neuroprosthesis” AND "tremor". We removed 1136 duplicates to yield 1260 records. We applied the following inclusion criteria for our review: (1) full-text journal articles or conference proceedings with complete introduction, methods, results and discussion sections, (2) investigation of any type of peripheral electrical stimulation applied to patients with ET or PD tremor, and (3) descriptions of the level of tremor reduction by means of electromyography, kinematics or clinical scales. We applied the following exclusion criteria: (1) systematic reviews, books and book chapters; (2) manuscripts describing purely mechanical devices to reduce tremor (e.g., exoskeletons, orthoses, gloves), drug or pharmacological-based treatment, or interventions directly at the brain level (e.g., DBS, FUS, transcranial magnetic stimulation, transcranial direct current stimulation, transcranial alternating current stimulation, etc.); (3) conference proceedings including case studies with only one participant, or studies completed on only healthy participants; (4) abstracts, posters, conference proceedings, or papers missing clearly described methods, results, or discussion sections; and (5) non-English papers.

**Fig. 1:**
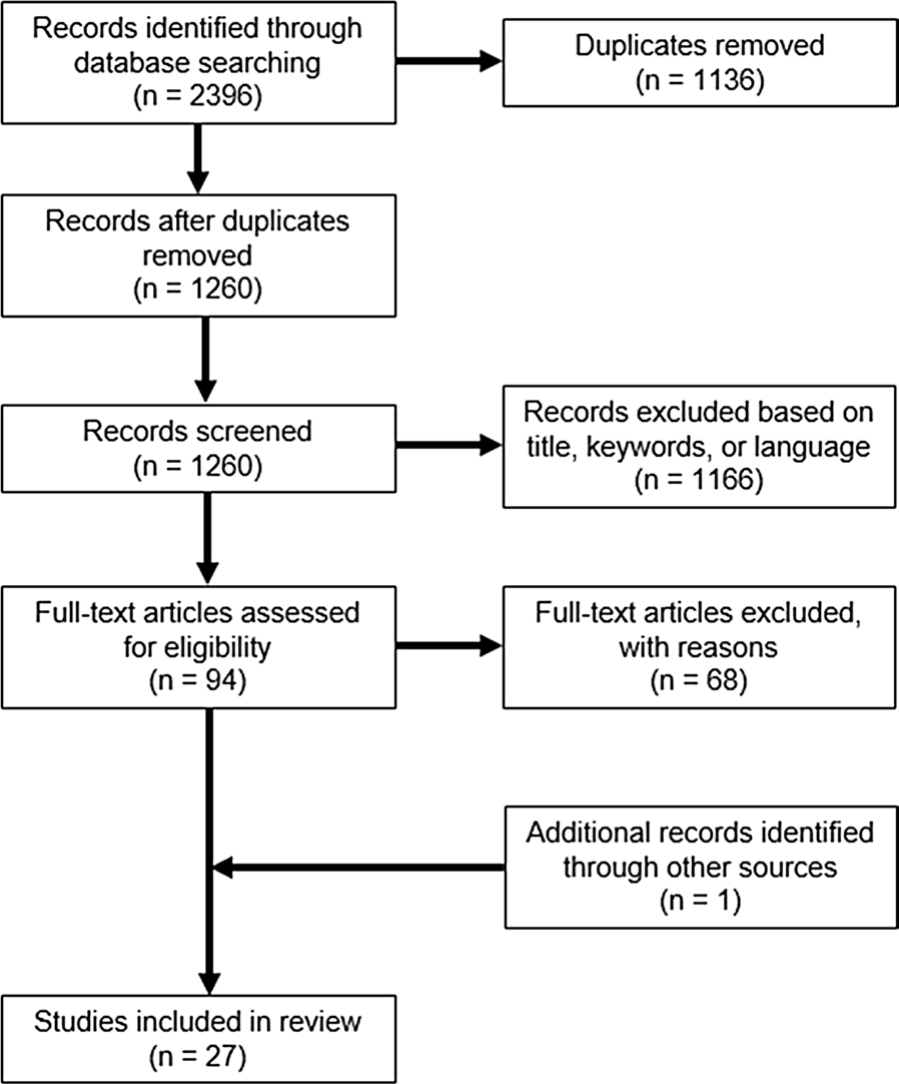
Flow diagram of screening process and identifying final papers included in this review

There were several studies that published both conference proceedings describing preliminary results and a following full-text journal article on the same study; the review team agreed to exclude the conference proceedings from this review and only include the journal articles. Additional searching was conducted on an as-needed basis, with one paper included [17]. As a result, from 1260 records, we identified 27 full-text journal articles meeting our inclusion and exclusion criteria that were considered for this review (Fig. 1).

## Results

Table 1 summarizes the 27 papers included in our review, noting the clinical population, strategy, stimulation parameters, tremor assessment method, main results, and implied physiological mechanism for each paper.

**Table 1:** Summary of the methodology and results from the studies reviewed

Article	Population	Strategy	Stim. location	Stim. pulse width [us]	Stim. frequency [Hz]	Stimulation protocol	Tremor assessment	Main results	Physiological mechanism
Bó et al. [20]	10 ET (moderate-severe)	FES: co-contraction	SF: heterogeneous wrist and finger muscles	150	40	10-50 s/trial, stim ON vs stim OFF, 5–7 trials	Kinematics, tremor power at wrist	Most significant acute tremor attenuation: 37.18%-94.68%	Increasing joint stiffness
Britton et al. [38]	10 ET; 9 PD, 8 HV	Single shock > MT	SF: median nerve	500	Single shock	Single shock	sEMG	Significant EMG reduction from 90 to 210 ms post stimulus	Afferences reset central tremor oscillators
Dideriksen et al. [42]	4 ET; 5 PD (mild-severe)	afferent < MT: out-of-phase EMG based	SF (2 ET, 3 PD), IM (2 ET, 2 PD): wrist flexors-extensors	400	100	150 s/trial, stim ON vs stim OFF, 20% and 40% DC, 10 trials	Kinematics, tremor power at wrist	Average highest acute reduction: 54 ± 20% (IM) and 50 ± 41% (SF)	Ia afferent fibers, reciprocal inhibition
Dosen et al. [16]	2 ET; 4 PD (mild-severe)	FES and afferent < MT: out-of-phase EMG based	SF: wrist/finger flexors-extensors	300	100	120 s/trial, stim ON vs stim OFF, 5 trials per modality	Kinematics, tremor power at wrist	Average acute reduction: 60 ± 14% (> MT) and 42 ± 5% (< MT) (p < 0.05)	Generation of opposite forces to tremor oscillations; Ia afferent fibers, reciprocal inhibition
Gallego et al. [39]	4 ET; 2 PD (mild-severe)	FES: co-contraction	SF: wrist flexors-extensors	250 or 300	30 or 40	30 s/trial; stim ON vs stim OFF, 6–12 trials	Kinematics, tremor power at wrist	Average acute reduction: 52.33 ± 25.48% (p < 0.05)	Increasing joint stiffness
Gillard et al. [29]	3 PD, 3 HV	FES: out-of-phase accelerometer based	SF: wrist/finger flexors-extensors	*	*	30 s/trial, stim ON vs stim OFF, 10 trials	Kinematics at finger/wrist	Average acute reduction: 84.50 ± 2.20%	Out-of-phase forces
Grimaldi et al. [17]	1 PD, 1 ET, 1 cerebellar syndrome	FES: co-contraction	SF: wrist/elbow flexors-extensors	100	30	~ 30 s/task, stim ON vs stim OFF, 15 trials (5 tasks)	Kinematics at finger/wrist/elbow; CNF-TES scale	Most significant acute tremor attenuation in one ET patient: ~ 50%	Increasing joint stiffness
Hao et al. [32]	8 PD (moderate)	afferent < MT: continuous (EMG triggered)	SF: radial nerve (dorsal skin of hand)	200	250	15 s/trial, stim OFF(5 s)- ON(5 s)-OFF(5 s), 9–13 trials	Kinematics and sEMG at finger/wrist/elbow flexors-extensors	Average acute reduction: 61.56 (kinematics across degrees of freedom); 47.97% EMG across degrees of freedom)	Cutaneous afferents and propriospinal interneurons
Heo et al. [21]	18 ET (moderate)	afferent < MT: continuous	SF: wrist/elbow flexors-extensors	300	100	15 s/trial, Pre-Stim ON-Post 5 min, 9 trials	Kinematics at finger/forearm/arm	Average acute reduction: 90% (finger), 58% (hand), -50% (forearm); Post 5 min: 88% (finger), 61% (hand), 27% (forearm)	Afferences might modulate supraspinal tremor oscillators
Heo et al. [22]	18 ET (mild-severe)	afferent < MT: continuous	SF: wrist/elbow flexors-extensors	300	100	Spiral drawing task, Pre-Stim ON-Post 5 min, 9 trials	Kinematics at finger/forearm/arm	Acute average reduction: 12%. Significant differences (p < 0.05) for Pre-Stim and ON-Post 5 min	Afferences might modulate supraspinal tremor oscillators
Heo et al. [34]	14 PD (mild-severe)	afferent < MT: continuous	SF: wrist flexors-extensors	300	100	15 s/trial, Pre-Stim ON-Post 5 min, 9 trials	Kinematics at finger/forearm/arm	Reduction in 50–71% of patients. Average acute: 68% (finger), 62% (hand), 53% (forearm); Post 5 min: 56% (finger), 59% (hand), 60% (forearm)	Afferences might modulate supraspinal tremor oscillators
Heo et al. [35]	14 PD (same Heo et al. [34]), 9 SWEEDs	afferent < MT: continuous	SF: wrist flexors-extensors	300	100	15 s/trial, Pre-Stim ON-Post 5 min, 9 trials	Kinematics at finger/forearm/arm	No reduction data. Only significant differences (p < 0.05) for Pre-Stim ON in PD. No reduction for SWEEDs	Afferences might modulate supraspinal tremor oscillators
Isaacson et al. [26]	263 ET (mild-severe)	afferent < MT: out-of-phase kinematics (open-loop)	SF: radial and median nerves at wrist	†	†	Clinical-trial: 3 months, 2 × 40 min stim session/day	TETRAS, select BF-ADL tasks, kinematics at wrist, CGI-I, PGI-I, QUEST	62% (TETRAS) and 68% (BF-ADL) of severe/moderate patients improving to mild/slight	Afferences modulate ventral intermediate nucleus
Javidan et al. [40]	3 ET, 4 PD, 6 cerebellar tremor	FES: out-of-phase kinematics based	SF: wrist, elbow flexors-extensors	100	30	20 min/trial, Stim ON vs Stim OFF, unknown number of trials	Kinematics at wrist	Average acute reduction at wrist: 73 ± 14% (ET), 62 ± 5% (PD), 62 ± 38% (cerebellar tremor)	Generation of opposite forces to tremor oscillations
Jitkritsadakul et al. [30]	34 PD (moderate)	> MT: continuous	SF: fingers APB, FDI, SDI	150	50	10 s/trial, Pre-Stim ON, 2 trials	Kinematics and sEMG at finger/forearm/arm; UPDRS	Average acute reduction (tremor power): 49.57 ± 38.89% (p < 0.05)	Afferences interfere with the cerebello-thalamo-cortical circuit
Jitkritsadakul et al. [33]	30 PD (moderate)	> MT: continuous	SF: fingers APB, FDI, SDI	150	50	10 s stim, Pre-Stim ON; 30 min session; Sham vs Stim	Kinematics at hand (glove); UPDRS; VAS	Average acute reduction (RMS, x-axes): 60.22 ± 38.85% (p < 0.05); significantly different from sham group	Afferences interfere with the cerebello-thalamo-cortical circuit
Kim et al. [25]	9 ET (moderate-severe)	afferent < MT: out-of-phase kinematics based and open-loop	SF: radial nerve at wrist	200	50, 100, 200	20 s/trial, stim. OFF (10 s)-ON (10 s); open-loop, closed-loop; 12.5%, 25% and 37% DC; 18 trials	Kinematics at wrist; TETRAS; qualitative assessment	Average acute reduction: 42.17 ± 3.09% (p < 0.05). No differences open vs closed loop	Not proposed
Lin et al. [23]	23 ET (moderate-severe)	< MT: out-of-phase kinematics (open-loop)	SF: radial and median nerves at wrist	300	150	Pre, 40 min stim, Post; Sham vs Stim group	TETRAS (spiral)	Average post reduction (TETRAS): 60 ± 8.4% (p < 0.05), significantly different from sham group	Afferences modulate ventral intermediate nucleus
Mones et al. [28]	5 PD	Single shock above MT	SF: ipsilateral and contralateral ulnar nerve at wrist	500	Single shock	Single shock	iEMG	No change in tremor amplitude. Change in tremor frequency after shock	Afferences reset central tremor oscillators
Munhoz et al. [18]	5 ET, 2 peripheral neuropathy	TENS > MT: continuous	SF: brachial plexus on neck, C7 spinous process	250	5, 10, 50, 100	Pre, 15 min stim, Post	Kinematics at wrist, WHIGET scale	No significant reduction	Wrong afferent fibers targeted or stimulation parameters
Muceli et al. [36]	1 PD	afferent < MT: out-of-phase EMG based	SF: wrist/finger flexors-extensors	200	100	60 s/trial, stim ON(30 s) vs stim OFF(30 s), 2 trials	Kinematics, tremor power at wrist	Acute reduction in one patient: 58%	Ia afferent fibers, reciprocal inhibition
Pahwa et al. [24]	77 ET (moderate-severe)	afferent < MT: out-of-phase kinematics (open-loop)	SF: radial and median nerves at wrist	300	150	Pre, 40 min stim, Post; Sham vs Stim group	TETRAS, select BF-ADL tasks, CGI-I scale	Average post reduction (task 4 TETRAS): 46% (stim group) different from 24% (sham group)	Afferences modulate ventral intermediate nucleus
Pascual-Valdunciel et al. [27]	11 ET (moderate)	afferent < MT: EMG based (SATS), continuous	SF: median and radial nerves at arm; IM: FCR and ECR	400 (SF), 200 (IM)	100	Pre; 60 s/trial, stim ON(30 s) vs stim OFF(30 s), > 6 trials, continuous/SATS; Post; Post24h	Kinematics at wrist/elbow/shoulder; FTM scale; contralateral arm	Average acute reduction at wrist: 32%; Post reduction after SATS-IM (6/6) patients	Ia afferent fibers, reciprocal inhibition, propriospinal system
Popovic et al. [41]	3 ET, 4 PD, 5 HV	FES: out-of-phase EMG based	SF: wrist, elbow flexors-extensors	250	40	approx. 60 s/trial, stim ON vs stim OFF**	Kinematics at wrist	Average acute reduction: 67 ± 13%	Generation of opposite forces to tremor oscillations
Spiegel et al. [37]	8 PD	> MT	SF: median (IP and CL) and ulnar (IP) nerves at wrist	200	2, 3, 5	approx. 316 s/trial, single shock, post 5 min, 4 trials without shock-4 trials with shock	sEMG	Tremor amplitude not reported. Change in tremor frequency after stimulation	Afferences interfere with central tremor oscillators
Widjaja et al. [19]	1 ET	FES: out-of-phase model based (EMG + kinematics)	SF: wrist flexors-extensors	200	25	40 s/trial, Stim ON vs Stim OFF, 1 trial	Kinematics at wrist	Individual acute tremor attenuation: 57%	Generation of opposite forces to tremor oscillations
Xu et al. [31]	2 PD (moderate)	afferent < MT: continuous (EMG triggered)	SF: radial nerve (dorsal skin of hand)	200	250	15 s/trial, stim OFF(5 s)-ON(5 s)- OFF(5 s), 9–13 trials	Kinematics and sEMG at finger/wrist/elbow flexors-extensors	Significant acute reduction compared to OFF (p < 0.05). No values provided	Cutaneous afferents and propriospinal interneurons

*ET* essential tremor, *PD* Parkinson's disease, *HV* healthy volunteer, *sEMG* surface EMG, *SF* surface stimulation, *IM* intramuscular stimulation, *BF-ADL* Bain and Findley ADL, *CGI-I, PGI-I* Clinical and Patient Global Impression scores, *QUEST* Quality of Life in Essential Tremor, *APB* abductor pollicis brevis, *FDI* first dorsal interossei, *SDI* second dorsal interossei, *SATS* selective and adaptive timely stimulation, *IP* ipsilateral, *CL* contralateral, *DC* duty cycle, *VAS* visual analog scale, *iEMG* intramuscular EMG.

*Not described in paper; **: ((3 s stim + 1 s pause)*3 + 9 s pause)*3 reps

† not described in paper but clinical study based on prior (Pahwa et al. [24]) study and most likely replicated those parameters

### Target populations

Among the review articles, ten studies reported the effects of peripheral electrical stimulation results on patients with ET [18–27] while ten studies reported on patients with PD [28–37] and seven studies included both patients with ET and patients with PD [16, 17, 38–42]. In order to compare electrical stimulation strategies, some studies also included healthy volunteers that either mimicked tremorgenic activity during experimentation or were subject to artificially induced tremors for experimentation [31, 38, 41]. Four papers reported additional results on other tremor conditions, including cerebellar ataxias [17], patients with scans without evidence of dopaminergic deficits (SWEEDs) [35], peripheral neuropathy [18], and multiple sclerosis [40]. For this review, we will focus on the ET and PD populations as the other conditions were excluded.

It is worth noting that the articles had a wide range of sample sizes, ranging from 1 to 263 [19, 26]. Articles with a relatively large sample size such as Lin et al. [23] included 23 patients with ET; Jitkritsadakul et al. [30, 33] included 34 patients with PD in 2015 and 30 patients with PD in 2017; Pahwa et al. [24] included 77 patients with ET; and Isaacson et al. [26] included 263 patients with ET in a multi-center clinical trial. The rest of the articles analyzed in this review had sample sizes of 20 patients or less. In some cases, some articles can be considered case reports as Widjaja et al. [19] or Muceli et al. [36] only included one experimental subject, and Xu et al. [31] only included two patients for experimentation, but were still included in this study due to the complete introduction, methods, results and discussion sections.

### Electrical stimulation approaches

Two main electrical stimulation approaches were used to manage tremor reduction: functional electrical stimulation (FES) and the stimulation of afferent pathways. The motor threshold, defined as the minimal electrical current intensity that evokes a muscle twitch, is a key feature to understand the differences between these electrical stimulation approaches [36]. Seven articles used FES above the motor threshold to elicit muscle contractions, which generate forces in the musculoskeletal system and, therefore, reduce tremor oscillations [17, 19, 20, 29, 39–41]. Alternatively, 16 studies applied electrical stimulation of afferent pathways below the motor threshold in order to neuromodulate the central nervous system [21–28, 31, 32, 34–38, 42]. Only one article compared the effects of FES versus stimulation of afferent pathways [16]. Three studies applied electrical stimulation above motor threshold with the purpose of recruiting afferent fibers, but muscle contraction was not the primary goal [18, 30, 33].

### Stimulation strategies

Electrical stimulation of muscles and peripheral nerves can be applied following different patterns based on mechanical and physiological models. The co-contraction strategy is based on the model that at a single joint, an agonist and antagonist muscle or group of muscles receive the tremorgenic input, producing involuntary mechanical oscillations [43]. In this strategy, simultaneous electrical stimulation applied above the motor threshold in both agonist and antagonist muscles increase the impedance of the joint and therefore minimize the involuntary oscillations [17, 20, 39]. However, the agonist–antagonist muscle pair commonly follow an out-of-phase activation pattern, as the muscles alternate contraction at the tremor frequency [43, 44]. Based on this behavior, the out-of-phase stimulation strategy stimulates the antagonist muscle when the tremorgenic agonist muscle is involuntarily activated [41]. This method has been applied for both FES and stimulation of afferent pathways. FES applied in an out-of-phase pattern produces opposite forces to the tremor oscillations [16, 19, 29, 40, 41]. To further refine the stimulation strategies by implementing closed-loop capability, neuromusculoskeletal models have been developed to characterize both tremorgenic and voluntary movements considering the mechanical properties of muscles and tendons, with neural circuitries and electrical stimulators acting as controllers of the muscle activation [45]. Closed-loop control algorithms allow real-time adaption of the stimulation to the tremor oscillations by making use of kinematics measurements, [29, 39–41], electromyography (EMG) signals of the target muscles [16, 27, 36, 42], or both measurement types [19]. One proposed model included electrophysiological information by means of electroencephalography and EMG recordings providing inputs to a neuromusculoskeletal model in order to isolate tremorgenic activity from voluntary movement and achieve adaptive joint impedance [46]. However, that study only involved one tremor patient as a case study for tremor reduction testing and, therefore, was excluded from this review.

**Fig. 2:**
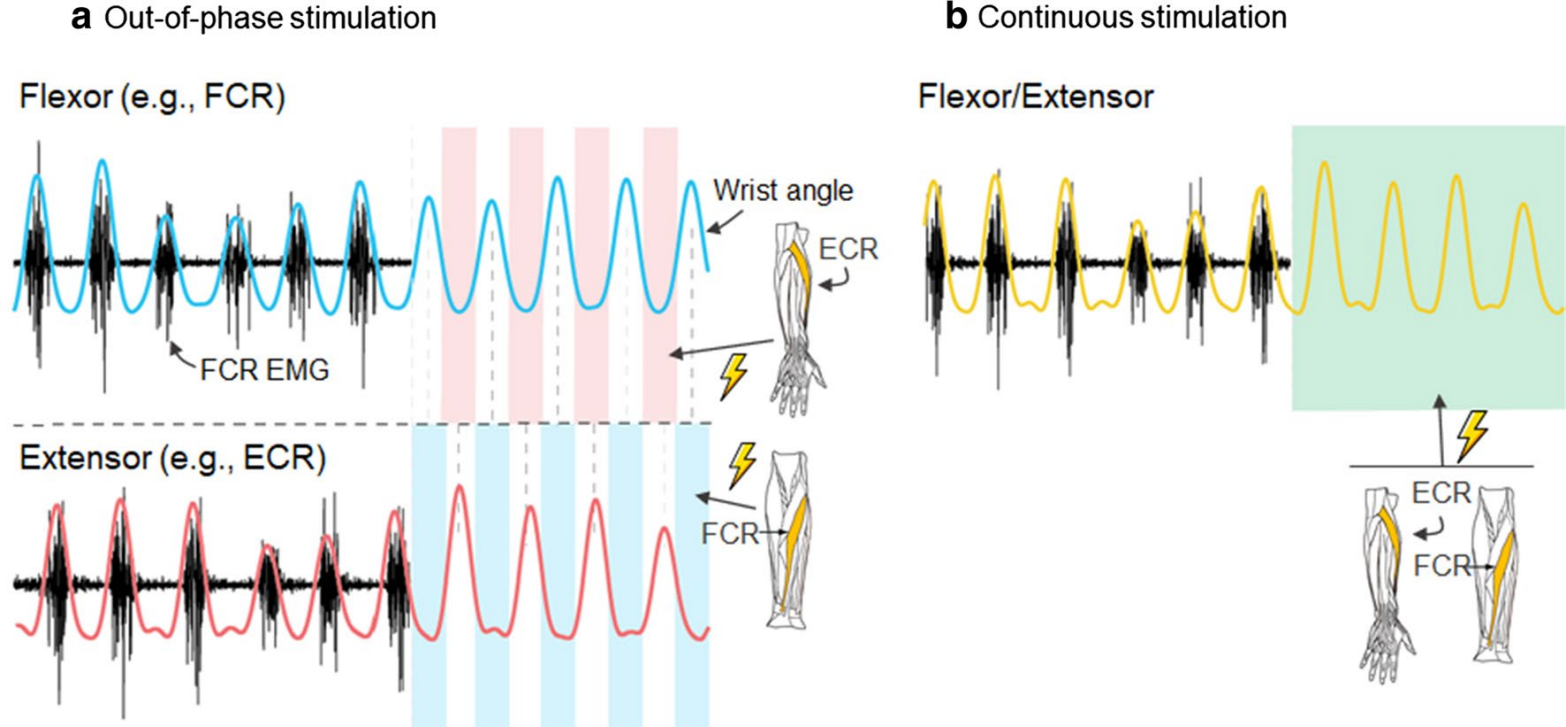
Stimulation strategies. **a** Out-of-phase stimulation. Tremorgenic bursts are detected in the recording window EMG (gray lines) and future tremorgenic bursts are predicted during the stimulation window (red and blue lines). Stimulation is then applied to the antagonist (red and blue colored rectangles) out-of-phase with respect to the activity in each target muscle during a stimulation window (adapted figure from Dosen et al. [16]). **b** Continuous stimulation. Electrical stimulation is applied during the entire stimulation window (green colored rectangle) without following any time pattern

Stimulating afferent pathways below motor threshold combined with the out-of-phase pattern demands precise stimulation timing with muscle activation. EMG-based algorithms have been used to drive the out-of-phase stimulation strategy, using sequential recording and stimulation windows to avoid the presence of artifacts in the EMG recordings [16, 36, 42]. EMG signals from the agonist–antagonist muscle pair are demodulated to extract the tremor frequency and period, which are used to predict the next tremor bursts and deliver the asynchronous stimulation based on an out-of-phase pattern (Fig. 2a). Pascual-Valdunciel et al. [27] adapted an out-of-phase strategy and applied it with stimulation below motor threshold: instead of predicting a group of tremorgenic bursts based on the demodulated tremor, they computed the root mean square (RMS) of short EMG windows (10 ms) in real-time. Then, if one or both RMS values from the recorded muscles exceeded an adaptive threshold, a short stimulation burst was delivered to the antagonist muscle. This enabled simultaneous stimulation of both muscles if co-contraction occurred.

Alternative strategies derived from the out-of-phase pattern have also been proposed in the last two years. Tremor frequency can be estimated by means of gyroscopes to drive an open-loop sequential stimulation of radial and/or median nerves [23, 24, 26]. This variation of the out-of-phase pattern preserves the synchronous stimulation at the mechanical tremor frequency and therefore avoids artifact issues with the EMG recordings. However, the activation of afferent pathways with the real physiological tremorgenic phase is not achieved.

Some studies applied electrical stimulation without following any time pattern or synchronization to mechanical or physiological events [21, 22, 28, 30–35]. This stimulation strategy has been labelled as continuous stimulation in this review (Fig. 2b). To limit stimulation during only tremorgenic activity instead of during voluntary movements, EMG recordings have been used to detect tremor activity and therefore enable continuous stimulation only when tremor is present [31, 32].

A small number of studies tested the effects of single shock electrical stimuli in tremor features, such as amplitude, frequency or refractory rate [28, 37, 38]. The purpose of these approaches was to study neurophysiological responses after electrical stimulation to explore possible neuromodulation outcomes, and as such are not aligned to those presented above to target exclusively tremor reduction.

### Stimulation parameters

**Fig. 3:**
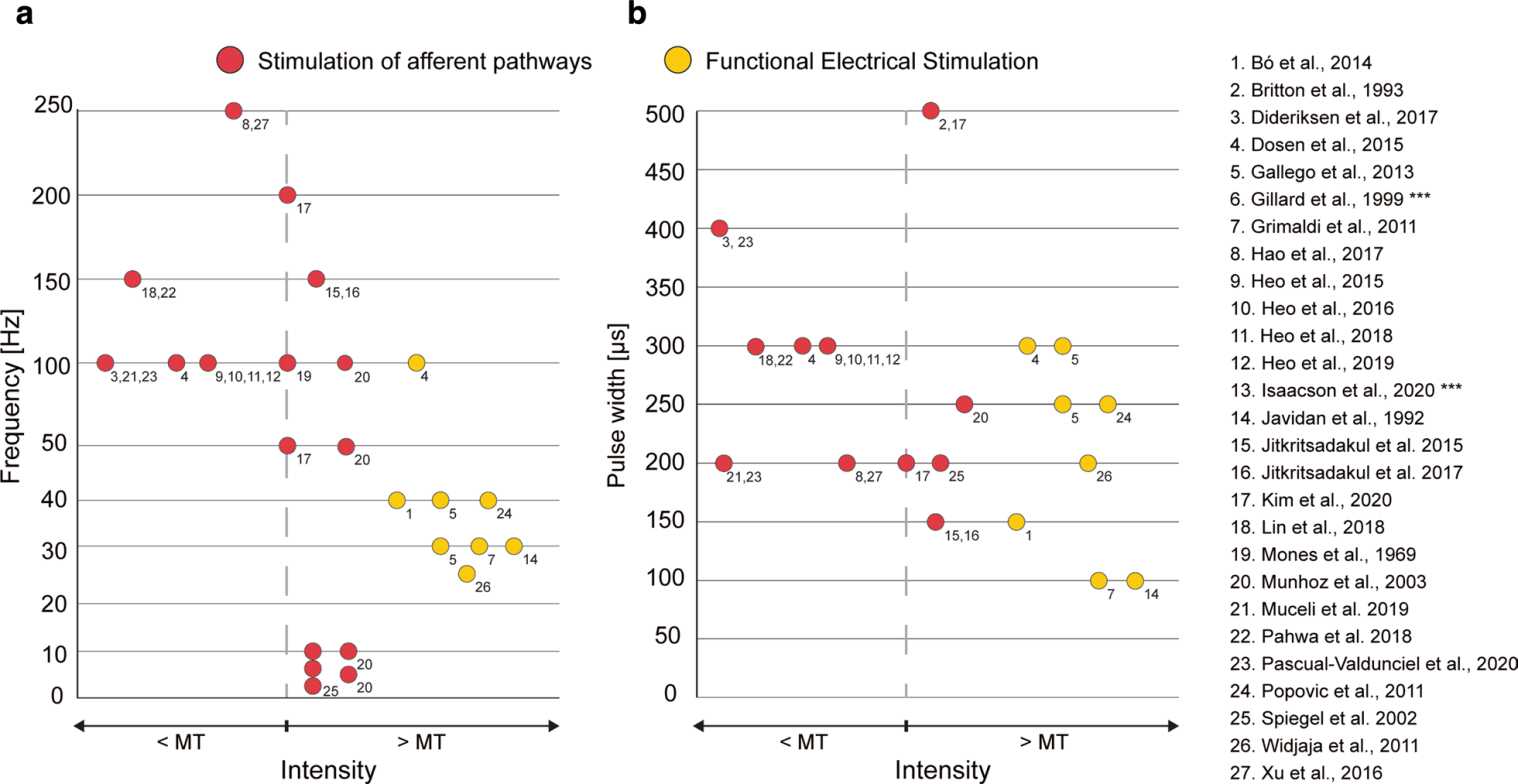
Stimulation parameter scatter plot. **a** Frequency vs. stimulation intensity. **b** Pulse width vs. stimulation intensity. Red dots represent studies using electrical stimulation of afferent pathways. Yellow dots represent studies using FES. Studies testing multiple stimulation parameters are represented by multiple dots. Studies performed by the same research group or replicating the same conditions are represented by the same dot. Note that four studies used stimulation of afferent pathways with stimulation intensity above motor threshold. ***Not described in paper

Stimulation intensity or amplitude (measured in mA) is a determining parameter to recruit muscle and sensory fibers. Absolute values were reported to vary from 0.3 mA in sensory intramuscular stimulation [27] to 36 mA in some FES experiments [17]. Commonly, stimulation intensity is normalized for each subject to their motor threshold, which is defined as the minimum current that invokes muscle movement, or their perception threshold, which is defined as the minimum current that the subject can perceive [36], and is also referred to as the radiation or sensation threshold [32]. Stimulation frequency was set in a range varying from 2 to 250 Hz (see Fig. 3a). Stimulation approaches using FES applied stimulation frequency between 25 [19] and 100 Hz [16]. Studies targeting afferent pathways used stimulation frequencies between 2 [37] and 250 Hz [31, 32]. There are two outliers for the minimum stimulation frequency, Spiegel et al. [37], who tested at 2 Hz, 3 Hz, and 5 Hz, and Munhoz et al. [18], who tested at 5 Hz, 10 Hz, 50 Hz, and 100 Hz. Certain studies have tested varying the stimulation frequencies while maintaining other stimulation parameters in order to determine optimal tremor reduction values [18, 25].

The pulse width is another stimulation parameter and is defined as the duration of each electrical stimulus applied. Across all of the reviewed studies, the pulse width ranged from 100 to 500 μs, with the maximum value of 500 μs used by studies applying a single electrical stimulus [28, 38] (see Fig. 3b). Values between 100 and 300 μs are reported for FES applications, while it seems that higher values ranging from 150 to 400 μs are used to stimulate afferent pathways.

The duty cycle, described as the time length comprising a train of pulses, is only indicated in some stimulation strategies [16, 23–25, 36, 42]. This parameter is typical for out-of-phase and derivative strategies, and it usually references a portion of the tremor period. For instance, Dosen et al. [16] fixed this parameter at 40%, which is equivalent to delivering a train of pulses during 80 ms if tremor frequency is 5 Hz. Lin et al. [23] and Pahwa et al. [24] applied a duty cycle equivalent to half of the tremor period. Dideriksen et al. [42] and Kim et al. [25] tested the effect of several duty cycle values: 20% and 40%; and 12.5%, 25% and 37%; respectively.

The stimulus waveform is a fifth parameter that varied among the papers. The most common stimulus waveform is the squared wave, which might be monophasic [21, 22, 28, 30, 33–35, 37, 38] or biphasic [16, 19, 23–25, 27, 31, 32, 36, 39, 41, 42]. Some studies did not report this parameter [17–20, 26, 29, 40].

### Stimulation electrodes and location

Different types of electrodes have been used to stimulate nerves and muscles across studies. Conductive pads attached to the surface of the skin, referred to as surface electrodes in this review, are widely used during muscle and nerve stimulation in all reviewed studies except three [27, 36, 42]. Additional file 1: Table S1 summarizes the various electrodes utilized by the studies in this review. It is noteworthy that Dideriksen et al. [42] first used intramuscular electrodes and compared their effect to surface electrode stimulation. They used a pair of Teflon-coated stainless steel wires inserted into the muscle belly with a hypodermic needle. Muceli et al. [36] tested the feasibility of thin-film intramuscular electrodes, with both EMG recording and stimulation contacts embedded in the same wire, which were later used by Pascual-Valdunciel et al. [27], comparing its performance against surface electrodes.

Tremor often manifests in the upper limbs, although it can occur in other parts of the body, and all the articles reviewed here applied electrical stimulation to upper limb structures. All the studies applied stimulation on just one arm, commonly the side most affected by tremor. Only Mones et al. [28] and Spiegel et al. [37] explored the effects of stimulating contralateral ulnar or median nerves, respectively, while Munhoz et al. [18] stimulated both sides of the brachial plexus. Stimulating the contralateral limb of interest was not completed by Pascual-Valdunciel et al., but they reported observations of the contralateral limb due to stimulation effects [27]. The studies that targeted stimulation of muscle belly commonly applied electrodes over the flexor and extensor muscles of the wrist [20, 27, 39, 42]. Since the anatomy of the forearm comprises a great array of superimposed muscles controlling the wrist and fingers, some studies also placed electrodes over both wrist and finger flexors and extensors [16, 20, 29]. Other studies targeted both the wrist and elbow joint, placing electrodes over biceps brachii and triceps brachii [17, 21, 22, 34, 35, 40, 41]. Jitkritsadakul et al. [30, 33] designed an intervention protocol and glove device to stimulate the abductor pollicis brevis as well as the first and second dorsal interosseous muscles. Alternatively, electrical stimulation of the nerves has been explored instead of stimulation of the muscle belly. The main nerves stimulated were the distal branches of the radial nerve at the hand [25, 31, 32], median nerve at the elbow [38], median and ulnar nerves at the wrist [37], median and radial nerves at the arm [27], or a combined stimulation of radial and median nerves in the same session through a wearable device at the wrist [23, 24, 26].

### Experimental design

Tremor is not a homogeneous condition to all patients or pathologies, and different etiologies and neurocircuitries involved may lead to different tremor manifestations, such as in ET and PD. Therefore, most of the experimental protocols involve patients performing a specific task or behavior to trigger their associated tremor type or measuring the effect of stimulation on the presentation of rest tremor in patients with PD [9]. If needed, patients may be engaged in distracting cognitive tasks to trigger or worsen tremor. Those studies including both patients with ET and patients with PD indicated different experimental tasks to account for the different tremor presentation based on clinical diagnosis.

The most common protocol for patients with ET involved delivering electrical stimulation while the participants kept their arms outstretched and unsupported against gravity [16, 17, 19–21, 27, 38, 40, 42]. In the experiments in patients with PD, participants were usually asked to keep their arms rested on a supported table [16, 29–37, 39, 41, 42]. Alternatively, Spiegel et al. [37] had their subjects with PD stretch their arms and hold perpendicularly to the body in a position in which postural tremor was maximal. Grimaldi et al. [17] had all of their subjects, which included participants with ET or PD, to perform kinetic tasks (e.g., finger-to-finger, or index finger-to-nose) while electrical stimulation was applied. Munhoz et al. [18] considered the application of electrical stimulation while the patient was performing functional tasks such as writing or drinking from a glass, Heo et al. [22] had patients complete the Archimedes spiral drawing task, and Kim et al. [25] had patients perform the bean transfer task.

All the studies included in this review applied peripheral electrical stimulation in a single session except one. Isaacson et al. [26] tested the effects of a wrist-worn wearable device in a clinical trial. Patients with ET were instructed to apply two 40-min sessions of stimulation per day over the course of three months. The status of the patients was assessed in three visits: at time of enrollment, one month after study onset, and study completion after three months. The majority of experimental protocols consisted of a single session acute comparison of baseline tremor periods with tremor periods while stimulation is applied (see Table 1). The stimulation time windows in these protocols varied from 5 to 30 s, and the number of trials per condition, if specified, ranged from one to 13. Some of the studies included comparisons between different stimulation strategies or conditions: Munhoz et al. [18] tested different stimulation intensities and frequencies; Spiegel et al. [37] tested different stimulation frequencies and nerves stimulated; Dosen et al. [16] benchmarked the effects of FES and afferent stimulation below motor threshold; Dideriksen et al. [42] tested the out-of-phase strategy and random-timed stimulation with two stimulation intensities and two duty cycles, using both surface and intramuscular electrodes; Kim et al. [25] assessed different combinations of frequency, duty cycle, and open/closed-loop control across trials; and Pascual-Valdunciel et al. [27] compared the effects of out-of-phase derived strategy and continuous stimulation, both applied with either surface or intramuscular electrodes. Only three protocols included a sham group to explore the possible placebo effect of stimulation on tremor reduction [23, 24, 33].

Conversely, prolonged effects after the application of peripheral electrical stimulation have also been explored by a limited number of studies. Hao et al. [32] and Xu et al. [31] explored the short-term tremor reduction effect immediately following 5 s after stimulation. Heo et al. [21, 22, 34, 35] assessed tremor before stimulation, during 15 s stimulation windows, and 5 min after stimulation ended. Lin et al. [23] and Pahwa et al. [24] proposed a 40 min stimulation session, assessing tremor before and after the stimulation, which was realized in the Isaacson et al. clinical trials [26]. Pascual-Valdunciel et al. [27] assessed tremor before the stimulation, while the stimulation was applied, after the stimulation, and 24 h after the experimental session.

### Efficiency of electrical stimulation to reduce tremor

The results of applying peripheral electrical stimulation are reported in most of our analyzed articles in terms of tremor reduction, namely, how tremor is reduced during or after stimulation is applied. Primary results corresponding to each study can be visualized in Table 1.

The most common method to assess tremor reduction in the studies reviewed is the use of kinematics measurements collected by means of accelerometers [18, 25, 26], gyroscopes [21, 22, 34, 35, 41], initial measurement units [16, 27, 36, 42] or motion capture systems [31, 32]. These sensors are placed onto the body segments targeted by stimulation, collecting kinematic data such as angle displacement, angular velocity, or acceleration that are offline analyzed using secondary metrics. As an example, Bó et al. [20] used the root mean square (RMS) of the tremor kinematics. Several groups [16, 27, 36, 42] computed the tremor power from the power spectrum density of the angle displacement in the tremor band (3–9 Hz) to assess the level of tremor reduction.

Quantitative evaluation of tremor reduction was, in a few cases, complemented or replaced by evaluation based on patient-reported or clinician-observed questionnaires or clinical scales rating the patient’s tremor status while performing postural, kinetic, or functional tasks [17, 18, 22–27, 30, 33]. Scales used in some studies were: The Unified Parkinson’s Disease Rating Scale (UPDRS) used to assess patients with PD [30, 33]; The Essential Tremor Rating Assessment Scale (TETRAS) [23–26] and Fahn-Tolosa-Marin Clinical Rating Scale (FTM) [22, 27]. In this line, Kim et al. [25] complemented the objective tremor reduction evaluation based on the tremor power spectrum density with a qualitative assessment based on a 7-item Likert-scale questionnaire; Jitkritsadakul et al. [30, 33] combined the use of RMS on different kinematics variables and the UPDRS tremor score to assess the effects of stimulation. In some cases, the effect of stimulation on tremor reduction was assessed based on the EMG signals collected at various muscles of interest [28, 31, 32, 38].

Articles that applied FES for tremor reduction reported variable results. Gillard et al. [29] achieved average acute reduction across three patients with PD of 84.5 ± 2.2%. Popovic et al. [41] reported 67 ± 13% average acute reduction in three and four patients with ET and PD respectively. Bó et al. [20] reported individual acute tremor reduction ranging from 37.2% to 94.7% in the RMS kinematics in ten patients with ET. While Grimaldi et al. [17] reported approximately a 50% improvement for one patient with ET in finger-to-finger tasks on a clinical scale (Clinical Neurophysiological Functional Tremor Evaluation Scale, CNF-TES) and kinematics power spectrum density, the same study reported no improvements for two patients with PD and cerebellar ataxia.

High variability in tremor reduction results was also found across studies applying stimulation of afferent pathways. Tremor reduction via below motor threshold stimulation was reported by Heo et al. in 2016 [22] averaging a 12% reduction in the RMS angular velocity at the distal finger segment and metacarpophalangeal joint of patients with ET during action tremors, yet Heo et al. in 2015 [21] reported a reduction of 90% and 77% RMS angular velocity at the same finger and metacarpophalangeal joints in postural tremors. Jitkritsadakul et al. [30] reported average acute tremor reduction in 30 patients with PD in both tremor power peak of 49.6 ± 38.9% and UPDRS from 10.6 (SD = 1.7) before stimulation to 8.9 (SD = 2.2) during stimulation. Similar tendencies in acute tremor reduction using continuous stimulation of afferent pathways was replicated in a sham study with 30 patients with PD, reporting 60.2 ± 38.9% average acute tremor reduction in the RMS of the wrist angular velocity, statistically higher than the sham group [33].

Only Dosen et al. [16] compared FES and stimulation of afferent pathways for tremor reduction, reporting an average acute tremor reduction of 60 ± 14% and 42 ± 5% for stimulation above and below motor threshold respectively. It is worth noting that even though on average stimulation above motor threshold outperformed stimulation below motor threshold, the statistical tests showed no significant differences between the stimulation levels.

Dideriksen et al. [42] explored the use of intramuscular electrodes in comparison with surface stimulation with the average of the highest acute reduction levels across all patients at 54 ± 20% (intramuscular) and 50 ± 41% (surface), respectively. Although results were not statistically different, the authors found that the variability across trials was lower with intramuscular stimulation, thus suggesting that intramuscular electrodes led to a more consistent effect on tremor reduction. This tendency was reinforced by Pascual-Valdunciel et al. [27], who reported intramuscular stimulation resulted in significant tremor reduction (32 ± 18%) compared to surface stimulation (6 ± 16%).

Results from different stimulation strategies are scarce and inconclusive. Dideriksen et al. [42] did not report differences in acute tremor reduction when applying out-of-phase strategy or random time stimulation, while Kim et al. [25] could not conclude that closed-loop stimulation achieved higher acute tremor reduction than open-loop stimulation. Conversely, Pascual-Valdunciel et al. [27] reported that synchronized stimulation with tremor activity achieved significant acute tremor reduction compared to continuous stimulation.

All studies reporting short-term tremor reduction after stimulation sessions used stimulation of afferent pathways [21–24, 26, 27, 34, 35]. Lin et al. [23] and Pahwa et al. [24] both reported patients with ET achieved higher TETRAS clinical scale scores after 40 min of stimulation compared to the sham group. Although these results might be promising, differences were only found in one item of TETRAS and no objective kinematics measurements were provided. This work was continued by Isaacson et al. [26], who reported that tremor was reduced in both kinematics and clinical scales when applying the same protocol in a three-month, open-label clinical trial. Additionally, Heo et al. reported a tremor reduction in kinematics during three stimulation trials (15 s per trial) and five min after stimulation, compared to baseline in patients with ET [21, 22] and PD [34, 35]. Pascual-Valdunciel et al. [27] reported tremor reduction immediately and 24 h after the intervention in some patients receiving synchronized stimulation with tremor activity.

### Physiological sources of tremor reduction

**Fig. 4:**
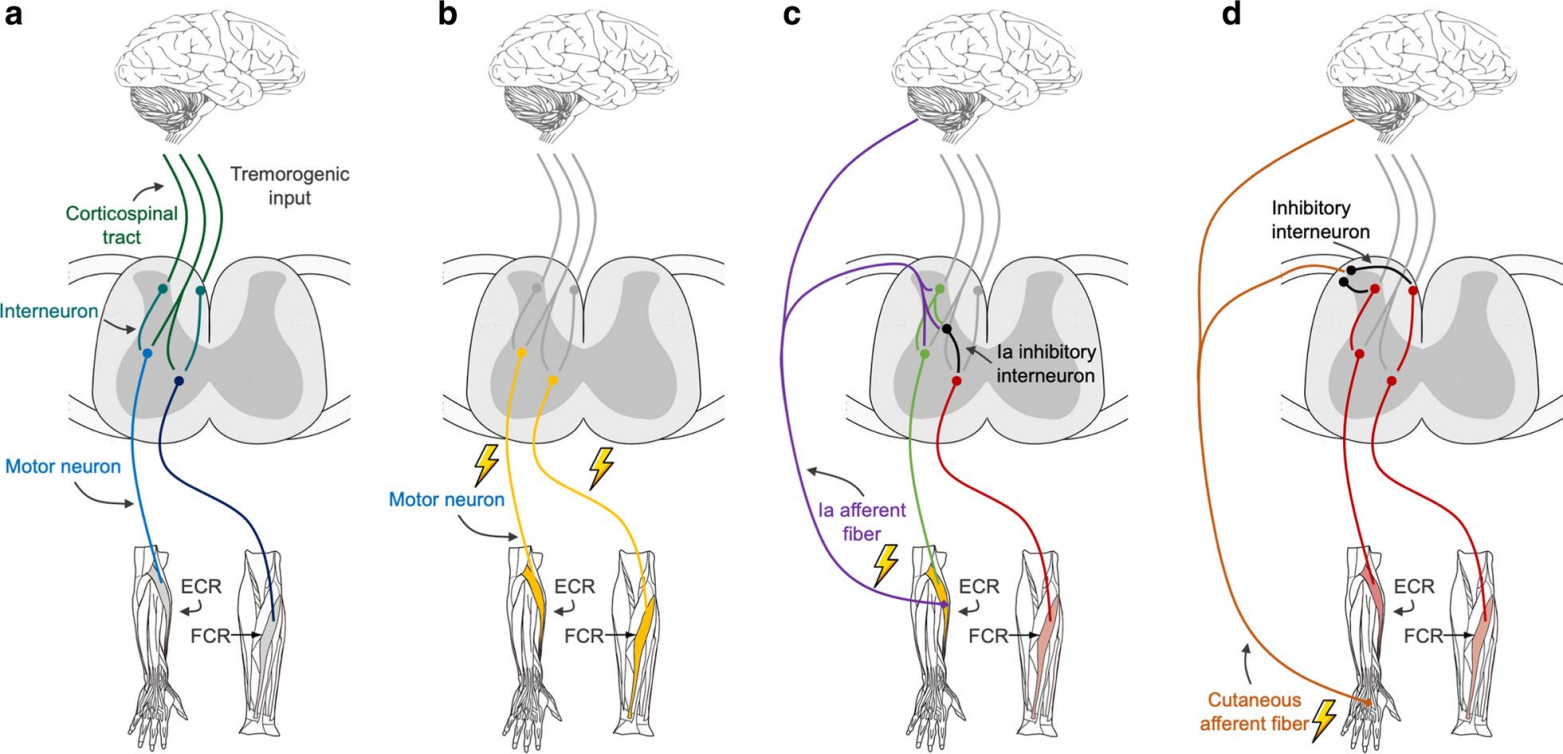
**a** The tremorgenic input is generated by a central brain oscillator and projected to the motor neuron pools monosynaptically via the corticospinal tract and disynaptically via interneurons. Several strategies to reduce tremors have been proposed: **b** functional electrical stimulation on the nerve or the muscle belly to elicit antagonist muscle forces and increase the joint impedance, **c** stimulation of Ia afferent fibers to decrease the excitability of the antagonist pool of motor neurons and alter the transmission of the tremorgenic input, and **d** stimulation of cutaneous afferent fibers to inhibit the interneurons and alter the disynaptic transmission of the tremorgenic input

Tremor reduction using peripheral electrical stimulation mainly involves two strategies: (1) FES, based on the generation of forces within the tremorgenic muscles to mechanically reduce tremors (Fig. 4b); and (2) the stimulation of afferent pathways, relying on the decrease in motoneuron excitability to alter the spread of the tremorgenic input from the central nervous system to the muscles (Fig. 4c and d). Figure 4a shows a schematic representation of the tremor generation.

FES takes advantage of electrical stimulation to recruit muscle fibers and evoke force [16, 17, 19, 20, 29, 39–41]. Specifically, the electrical current activates axons that generate action potentials and force once the action potential reaches the muscle fibers [47]. The higher the stimulation intensity, the greater the number of fibers recruited and therefore the higher the force produced. The co-contraction strategy achieves a reduction of tremor oscillations by means of stimulating a pair of agonist–antagonist muscles, evoking opposite forces which increase the joint impedance [20, 39]. The studies applying the out-of-phase strategy evoked antagonist forces to the tremorgenic muscle to increase joint stiffness, alternating the stimulation of tremorgenic agonist muscle and corresponding antagonist muscle [16, 19, 40, 41]. These studies also reported a risk of muscle fatigue and movement control alteration.

Alternatively, low current stimulation is also effective in activating excitatory postsynaptic potential from different sensory pathways that provide feedback to different circuitries at the central nervous system level [48]. The Ia afferent fibers have a lower rheobase than motor fibers, resulting in selective recruitment when stimulation intensities are applied below the motor threshold. Ia afferent fibers drive sensory information from muscle spindles, which sense the change in muscle fiber length, and make excitatory projections to the homonymous motoneuron and to an inhibitory interneuron of the antagonist muscle at the spinal cord, among others [43, 49]. This neural circuitry, responsible for the stretch reflex and reciprocal inhibition, has been exploited through the out-of-phase stimulation pattern to reduce the excitability of the motoneuron pool in a pair of antagonist tremorgenic muscles [16, 27, 36, 42].

In addition, Hao et al. [50] proposed that the supraspinal tremorgenic inputs are transmitted to muscles through the propriospinal system in PD, and indicated a potential role of propriospinal premotor interneurons that interact with afferent fibers, including cutaneous afferents. The propriospinal premotor interneurons receive input from cutaneous afferents and can modulate the excitability of the motoneurons in addition to monosynaptic corticospinal tracts [51, 52]. Continuous electrical stimulation of the cutaneous branch of the radial nerve might evoke disynaptic inhibitory postsynaptic potentials on the propriospinal neurons, and thus increase the recruitment threshold of motoneurons [32, 50].

Mones et al. [28] and Britton et al. [38] explored the effect of peripheral nerve single shock on tremor, suggesting the afferences could reset the central tremor oscillators, but they did not provide further discussion on the specific structures targeted nor the pathways activated. Spiegel et al. [37] explored the effect of low-frequency nerve stimulation on tremor frequency, but also did not provide any evidence on the afferent pathways activated, and only speculated about the alteration of thalamic neurons implied in tremor generation.

Interestingly, some studies have found a lasting effect of electrical stimulation on tremor reduction after the end of the stimulation session [21–27, 34, 35]. This implies that tremor generation at the supraspinal level or tremor transmission at the spinal level remain altered even after electrical stimuli is discontinued. Heo et al. [21, 22] suggested that the continuous feedback signal through the afferent pathways might alter the cerebrum and other possible sources of tremor at the central nervous system in ET. Regarding PD, Heo et al. [34, 35] and Jitkritsadakul et al. [30] proposed that continuous stimulation might reduce the hyper-excitability of the cerebello–thalamo–cortical circuit, which may be related to the impaired cerebellar inhibition in PD. Pahwa et al. [24] and Isaacson et al. [26] supported this idea, indicating that stimulating sensory pathways at the tremor frequency might disrupt tremorgenic activity in the ventral intermediate nucleus of the thalamus, which is a primary target of DBS for tremor reduction [23, 24].

## Discussion

Recent studies demonstrate the potential and usability of peripheral electrical stimulation as an intervention to reduce tremor. However, results are highly variable across studies and patients, which points out the need for consensus and standardized procedures to allow more reproducibility and cross-comparisons. There is substantial opportunity with peripheral electrical stimulation for tremor reduction interventions, as closed-loop control strategies allow a more precise delivery of the intervention in comparison to medications that can have broad central nervous system or bodily effects and to DBS or FUS that can be more invasive or irreversible, respectively; in addition, peripheral electrical stimulation can be discontinued at any time.

Several limitations of the reviewed studies have been identified: small sample sizes, lack of control or sham groups, varied stimulation parameters across studies, and in some, combined ET and PD groups. Also, the reporting of results was not always representative of the overall efficiency of the stimulation strategy (i.e., some studies report the highest reduction results for a subset of patients or single trials) and was highly variable among studies. As there is limited research on this topic, future study can address these issues as well as the long-term effects of the application of peripheral electrical stimulation.

Studies have been conducted mostly in two main pathologies: ET and PD. The pathophysiology of each of these disorders differs and, as a result, observable tremor reduction may be attributed to the modulation of different underlying tremor mechanisms. Some studies did not explicitly separate results between ET and PD subjects, implying that these authors may have expected similar responses to the tremor reduction strategy despite the different pathophysiology. Therefore, the inclusion of different pathologies in the same study using similar experimental procedures may limit conclusions drawn on the effectiveness of peripheral electrical stimulation and what mechanisms underlie the observable changes. Alternatively, this may lead to new hypotheses about similarities and differences of tremor reduction mechanisms in these disorders. Future studies examining relationships between diagnosis (ET, PD) and tremor response to stimulation using larger sample sizes and potentially coupled with additional physiological measures may help elucidate this issue.

Until 2015, most of the studies reported here applied current amplitudes that would elicit muscle contraction (i.e., stimulation above motor threshold) via FES. Most of the studies in this review applied electrical stimulation using strategies developed in previous groups’ contributions [53–56]. These models are commonly driven by control algorithms fed by kinematics and/or EMG data from the targeted limbs. Open-loop algorithms, which define a pre-calibrated stimulation pattern, might not be suited to fluctuating tremor status and could have limited efficacy compared to closed-loop control strategies, which show higher compliance to the tremor features [23–26]. On the other hand, EMG-based strategies, mainly used by the out-of-phase strategy, allow for superior time and spatial resolution, since the stimulation output can be synchronized with physiological tremorgenic activity from a single muscle, a desirable feature when stimulating afferent pathways. The stimulation artifacts present in the EMG signals still entails a challenge, despite some groups developing artifact suppression methods in the EMG [19], or using sequential recording and stimulation windows [16, 27, 36, 42]. Finally, kinematics-based strategies allow real-time control based on limb relative position and avoid dealing with stimulation artifacts, but in exchange have inferior time and spatial resolutions, since the stimulation cannot target specific muscle movement or electrophysiological activity due to the electromechanical delay [19, 25]. The use of neuromusculoskeletal models that are able to isolate voluntary movements from tremor oscillations were not indicated in the afferent stimulation studies. The deployment of these models integrated with both kinematics and electrophysiological signals could enhance the delivery of electrical stimulation of afferent pathways and thereby overcome the limitations of previous stand-alone approaches.

Stimulation parameters such as frequency, pulse width, and waveform play a role in tremor reduction, but are still to be determined. These parameters are known to have an effect on afferent stimulation efficiency [57]. However, there is high variability in these stimulation parameters across different studies, which highlights the need for more research on this area. Stimulation frequencies lower than 50 Hz are used in FES applications to minimize muscle fatigue, while frequencies between 50 and 250 Hz are preferred for recruiting afferent fibers with low amplitude stimulation. FES uses high stimulation intensities to elicit muscle fiber contraction of a sufficiently large population to produce the desired forces opposing tremor. Higher stimulation frequencies are reported to rapidly increase fatigue, and therefore limit this value for FES applications [58]. Conversely, stimulation of afferent pathways allows higher stimulation frequencies with lower stimulation intensities due to the lower rheobase of sensory fibers [48]. A similar division of pulse width parameter was observed. Shorter pulses were preferred for FES applications while longer pulses were preferred for stimulation below motor threshold. Regarding stimulus waveform, biphasic pulses are preferred to restore charge balance in tissues and minimize adverse effects such as skin irritation, but some studies applied monophasic pulses, which could lead to a limited application for a prolonged period of stimulation [58]. Finally, another critical step towards standardizing stimulation strategies is reporting the intensity of stimulation applied. This should be normalized either to the motor threshold or the perception threshold. Once some of these limitations are addressed, more comparisons among studies can be done and more conclusions can be drawn, not only on acute effects, but also on possible prolonged or longitudinal effects.

Surface electrodes are the preferred interface to deliver peripheral electrical stimulation as they are non-invasive, cost-effective and convenient to replace. However, their selectivity for targeting different structures can be limited since skin movement can adversely shift the stimulation location. Also, the variation of electrochemical properties of the electrode interface and current distribution could lead to an undesirable stimulation effect over time (e.g., impedance increase due to the gel drying out) [36]. Only three studies tested and presented results using intramuscular electrodes. Although transcutaneous or intramuscular electrodes are invasive and stimulate a reduced volume of tissue, they provide a more repeatable and robust outcome due to targeting the same group of fibers during stimulation and are less affected by the movement of the skin [36]. More research is needed regarding intramuscular electrodes to provide evidence about their safety and advantages against surface electrodes in long-term use to overcome the issue of their minimal invasiveness.

The majority of studies targeted tremor at the wrist joint, and only a few studies additionally stimulated muscles or nerves controlling the elbow or shoulder. However, mechanical tremor oscillations have been proved to propagate from proximal to distal joints [59]. Therefore, focusing on just one isolated joint might be insufficient to efficiently reduce tremor, as the tremor assessed at the wrist could be a product of the oscillations produced at the elbow or shoulder. The large variety in electrode locations targeting different groups of muscles or nerve branches, compounded by the limited number of studies and poor methodology descriptions in some cases, interfere with the reproducibility and comparison of results across studies.

Experimental design by some studies specified the age, sex, and duration of tremor for their subjects, but other studies did not report demographic or clinical details. Further examination of the relationships of demographics, disease-related features (e.g., duration, concomitant treatments such as medications) and other motor symptoms to stimulation response may be needed to better understand the clinical application of these stimulation strategies. For example, in a gradually progressive neurodegenerative disease such as PD, one may need to investigate whether the efficacy of the stimulation strategies change as the disease progresses, or if modifications in the stimulation techniques will be needed. Next, the posture held by each patient during stimulation trials should also be adapted according to the tremor pathology. However, some studies do not report the posture maintained by patients during stimulation trials; others used the same task (postural or rest) for different clinical populations, which would affect the tremor presentation and triggers. Only a few studies tested the effects of stimulation during basic functional tasks such as touching a finger to the nose [17] or drawing a spiral [22], perhaps due to the challenge of targeting tremorgenic oscillations instead of voluntary movements by control algorithms. Despite the difficulty, we propose that both intervention and assessment during activities of daily living could contribute to testing the efficacy of the peripheral electrical stimulation approach in real-life applications in order to determine or refine the strategy to minimize any effect on normal voluntary movement.

While some studies have proved that changes in muscle tone and reflexes are present in patients with PD as a motor control abnormality, few papers indicated quantitative muscle tone or reflexes assessment prior to, during, or after interventions [60–62]. Only Jitkritsadakul et al. [30] examined UPDRS scores including items for rigidity rated by the clinician or researcher administering the scale, and reported correlations between rigidity and various tremor aspects. Future studies including these measurements would provide additional knowledge on the mechanisms and effects of the different stimulation strategies.

Fourthly, only three studies included a control/sham group, which is an important component to test the efficiency of the experiments [23, 24, 33]. To address the lack of control groups, some studies have used different stimulation conditions across sessions [18, 25, 27, 37, 42]. These studies blinded subjects to the different stimulation strategies in order to observe which type of stimulation strategy leads to optimal tremor reduction. Finally, it is important to emphasize that all the studies in this review tested subjects during a single session except the clinical trial study presented by Isaacson et al. [26]. Meunier et al. [63], while excluded from this review due to investigating an alternate clinical population with writer’s cramp, completed a longitudinal study of TENS sessions spanning 2 weeks to determine if there was an effect on primary writing tremor at 5 Hz, 25 Hz, and 50 Hz, but reported that they observed either no effects or negative effects. The longitudinal approach should be of consideration for future studies, and in particular could influence the shift of acute tremor interventions to the development of therapeutic approaches applied in the home setting, such as done in the study by Isaacson et al. [26] where continuous accelerometer recordings were collected during each home therapy session to allow monitoring of progress without in-person clinician intervention or office visits.

### Physiological sources of tremor and reduction mechanisms

Most studies in the review hypothesized that stimulation acutely reduces pathological tremor. FES generates muscle contraction to counteract tremor, either in co-contraction or in an out-of-phase manner. The efficiency of the strategy depends on the accuracy of the control algorithm used to synchronize the timing and level of force produced by the stimulated muscles with the tremorgenic input [27]. This type of stimulation above motor threshold seems to achieve higher tremor reduction levels [16] although its inherent disadvantages like inducing fatigue and interfering with natural movements represent significant obstacles towards usability.

Stimulation of afferent pathways below motor threshold has been explored in the past five years as an alternative without the main drawbacks of stimulation above motor threshold [61]. Due to the early stage of research on this topic and the absence of precise knowledge about the neural circuitries implied in tremor generation, only hypotheses have been proposed to explain the tremor reduction outcomes. Important clues to improve protocols for stimulation below motor threshold at the peripheral level have been given by simulation studies. In ET, the Ia afferents can provide significant tremor input due to passive stretch, which shows an interplay between supraspinal input and spinal afferents in tremor generation mechanisms [43]. Additionally, with Ia afferents innervating an antagonist muscle pair, the counter-balancing of homonymous muscle excitability should be taken into account for analysis. By decreasing the excitability of the antagonist muscles receiving tremorgenic input and increasing the excitability of the homonymous muscle, this could modulate the tremorgenic activity [42]. However, tremorgenic patterns in patients with ET are not constant over time, as there can be in-phase and out-of-phase activation of antagonist muscles [44]. Dideriksen et al. [42] compared intramuscular and surface stimulation and did not find significant differences in tremor reduction between strategies. Instead, Pascual-Valdunciel et al. [27] reported significantly higher tremor reduction using intramuscular stimulation compared to surface stimulation. Both findings could indicate that cutaneous afferents (activated with surface stimulation) do not play a key role in tremor reduction compared to Ia afferent fibers (activated with intramuscular stimulation), particularly in ET. However, the variability of tremor reduction and reduced sample sizes must be kept in mind when interpreting these results [27, 42].

The model presented by Hao et al. [50] in PD proposes that the propriospinal premotor interneurons are involved in the transmission and processing of cortical tremorgenic signals. It is noteworthy that additional pathways could be activated with electrical stimulation (e.g., Ib afferent fibers) but the lack of sensitivity of EMG measurements in tremor management studies prevent the precise observation of each pathway. Selectively timed activation of Ia afferents and cutaneous afferents through electrical stimulation in synchronization with tremorgenic electrophysiological activity should be further explored to better understand and exploit spinal reflex mechanisms and inhibitory pathways for tremorgenic activity reduction [16, 27, 36, 42]. It is worth mentioning that one study, out of the scope of this review, proposed mechanical vibration as an alternative method to acutely suppress tremor via stimulation of afferent fibers [64]. No tremor reduction was achieved for the patients with ET, which might imply that electrical stimulation is more suitable than mechanical vibration to selectively activate afferent fibers interacting with tremorgenic circuitries.

Unveiling possible supraspinal mechanisms and eventually identifying any prolonged plastic effects will be of special importance. Thus, once the activated afferent fibers transmit signals to the spinal cord, the excitatory and inhibitory post synaptic potentials might disperse to both intraspinal networks and ascending pathways to finally reach the supraspinal centers involved in tremor generation, inducing neuromodulatory effects that are maintained over some period of time. Explanations suggested by those studies reporting a short-term effect on tremor reduction after stimulation (e.g., no longer than five min after stimulation) require further hypothesis generation and data to fully understand the conclusions on said effects. We propose that more studies should follow the line of Mones et al. [28] or Britton et al. [38], to focus on characterizing how the stimulation of the different afferent pathways interact with different neural structures involved in tremor generation to better design stimulation strategies tailored to each pathology.

In addition, characterizing the motor threshold is critical. Kim et al. [25] and Spiegel et al. [37] did not explicitly report their motor threshold level in reference to their stimulation levels, and therefore we cannot decisively infer that their results can all be attributed to stimulating afferent pathways. Similarly, Jitkritsadakul et al. [30, 33] and Munhoz et al. [18] state they stimulated above the motor threshold yet claimed that afferent pathways were exploited to reduce tremor; a main advantage of stimulating afferent pathways is avoiding muscle contraction and the resulting muscle fatigue, and as such their approach minimizes this advantage.

One important step for a better understanding of results would be the standardization of assessment metrics. Some papers use kinematics (usually with accelerometers or gyroscopes) while others only report clinical scales. Clinical scales are liable to inter- and intra-assessor variability and subjective judgment [65]. On the other hand, they can be used to assess functional tasks and measure the impact of therapies on activities of daily living, which cannot always be assessed by kinematics. Hence, reporting kinematics and functional scores together might benefit future comparisons across studies. The majority of studies also presented limited sample sizes and high variability in results. Few studies included statistical tests to support the tremor reduction data, and some of them presented only the highest suppression results for individual trials or patients. We highly recommend that results should include average results across groups or patients, and report statistical tests when possible, limiting individual case studies which have been proven to skew comparisons with their high variability.

### Methodological considerations of our review

This comprehensive review has several strengths. The literature search was conducted in four databases: Scopus, Embase, PubMed, and IEEE Xplore, and we used several screening iterations to select the final papers considered in this review. We do acknowledge, however, that this review also may present some limitations. Synthesizing results from studies that investigated electrical stimulation to reduce tremor in different disorders with differing pathophysiologies, and with very different stimulation parameters, may have its challenges, but we also note which studies pertain to ET, PD, or both ET and PD. Additionally, the main goal of this review was to ascertain the evidence that this approach can be actually explored as an alternative to classic tremor interventions, regardless of ET or PD diagnosis. While we decided to exclude all conference proceedings including case studies involving one subject, as well as contributions that included only methodology descriptions or sample sizes used in other contributions to improve the robustness of our review, it is possible that these publications could provide additional insights.

## Conclusions and future directions

Electrical stimulation below motor threshold stands as a promising intervention to manage pathological tremor due to its minor adverse effects compared to FES. Usability of peripheral electrical stimulation for regular and/or daily use to reduce pathological tremor has been showcased in two novel wrist-worn peripheral nerve stimulation devices [23, 24, 26]. More sophisticated wearable devices and algorithms should be pursued, especially combining both EMG and kinematic based-control to disregard voluntary movement components while reducing tremorgenic activity as well as the potential to focus stimulation at multiple muscles or joints of the tremorous limb. The development and testing of implantable technologies using intramuscular electrodes controlled with external wireless devices could also serve as a long-term solution [66, 67]. The preliminary evidence of prolonged tremor reduction after stimulation also opens the scope to develop longitudinal interventions towards a standardized therapy widely accessible to patients unresponsive to medication or ineligible for surgical treatments. Finally, it remains necessary to characterize the tremor reduction effects specific to tremor pathologies to personalize these strategies with PD patients, ET patients, or ET patients who later develop PD.

## Supplementary Information

**Additional file 1: Table S1.** Electrode types utilized by the studies included in this review.

## Data Availability

Not applicable.

## Abbreviations

DBS: Deep brain stimulation
EMG: Electromyography
ET: Essential tremor
FES: Functional electrical stimulation
FUS: High-intensity focused ultrasound
PD: Parkinson’s disease
RMS: Root mean square
TENS: Transcutaneous electrical nerve stimulation
