# Supplementary material for: Peripheral electrical stimulation to reduce pathological tremor: a review
Source: J Neuroeng Rehabil. 2021 Feb 15;18:33. doi: 10.1186/s12984-021-00811-9 (PMC7885254; doi:10.1186/s12984-021-00811-9)
Supplement: Supplementary file 1 — Additional file 1: Table S1. Electrode types utilized by the studies included in this review. [file 12984_2021_811_MOESM1_ESM.docx]

| **Table S1.** Electrode types utilized by the studies included in this review | |
| --- | --- |
| **Article** | **Electrode** |
| Bó et al., 2014 | Self-Adhesive: round ø3.2cm (smaller for forearm) |
| Britton et al., 1993 | * |
| Dideriksen et al., 2017 | Self-Adhesive: round ø3.2cm ; Intramuscular: ø0.5mm pair of Teflon wires |
| Dosen et al., 2015 | Self-Adhesive: 5x5cm (wrist); round ø3.2cm (wrist) |
| Gallego et al., 2013 | Multichannel surface array |
| Gillard et al., 1999 | Self-Adhesive: 4.5x4.5cm |
| Grimaldi et al., 2011 | * |
| Hao et al., 2017 | Self-Adhesive: round ø2.5cm |
| Heo et al., 2015 | Self-Adhesive: 5x5cm |
| Heo et al., 2016 | Self-Adhesive: 5x5cm |
| Heo et al., 2018 | Self-Adhesive: 5x5cm |
| Heo et al., 2019 | Self-Adhesive: 5x5cm |
| Isaacson et al. 2020 | Self-Adhesive: 2.2x2.2cm |
| Javidan et al., 1992 | Self-Adhesive: 2x3cm; Moistened pads |
| Jitkritsadakul et al. 2015 | Self-Adhesive: 5x5cm |
| Jitkritsadakul et al. 2017 | Self-Adhesive: 5x5cm |
| Kim et al., 2020 | Self-Adhesive: round ø2.5cm |
| Lin et al., 2018 | Self-Adhesive |
| Mones et al., 1969 | * |
| Munhoz et al., 2003 | Self-Adhesive |
| Muceli et al. 2019 | Thin-film double-sided intramuscular electrodes |
| Pahwa et al. 2018 | Self-Adhesive: 2.2x2.2cm |
| Pascual-Valdunciel et al., 2020 | Self-Adhesive: round ø3.2cm; Intramuscular: Thin-film double-sided |
| Popovic et al., 2011 | Self-Adhesive: round ø3.2cm |
| Spiegel et al., 2002 | * |
| Widjaja et al., 2011 | Self-Adhesive |
| Xu et al., 2016 | Self-Adhesive: round ø2.5cm |
| *: No description provided about the stimulation electrode. | |

**Additional Information**

***Search query***

Databases: Scopus, Embase, PubMed, and the Institute of Electrical and Electronics Engineers (IEEE).

Title OR abstract fields searched: "electrical stimulation" OR "electrical*" OR "nerve stimulation" OR "neuromodulation" OR "muscle stimulation" OR “neuroprosthesis” AND "tremor"

***Full inclusion and exclusion criteria***

**Inclusion criteria:**

1. Full-text journal articles or conference proceedings with complete introduction, methods, results and discussion sections.
2. Investigated any type of peripheral electrical stimulation applied to patients with ET or PD tremor.
3. Describing the level of tremor reduction by means of electromyography, kinematics or clinical scales.

**Exclusion criteria:**

1. Systematic reviews, books and book chapters.
2. Manuscripts describing purely mechanical devices to reduce tremor (e.g., exoskeletons, orthoses, gloves), drug or pharmacological based treatment, or interventions at the central nervous system level (e.g., deep brain stimulation (DBS), focused ultrasound (FUS), transcranial magnetic stimulation, transcranial direct current stimulation, transcranial alternating current stimulation, etc.)
3. Conference proceedings including case studies with only one participant, or studies completed on only healthy participants.
4. Abstracts, posters, conference proceedings, or papers missing clearly described methods, results, or discussion sections.
5. Non-English papers.
